# Supplementary material for: Resolving the full spectrum of human genome variation using Linked-Reads
Source: Genome Res. 2019 Apr;29(4):635–45. doi: 10.1101/gr.234443.118 (PMC6442396; doi:10.1101/gr.234443.118)
Supplement: Supplemental Material [file supp_gr.234443.118_Supplemental_longranger-2.2.zip › longranger-2.2.tar.bz2!/lr2.2-public/lib/stan/cmdstan/stan/doc/api/html/structstan_1_1gm_1_1write__csv__visgen-members.html]

Stan: Member List


|  |  |
| --- | --- |
|  | Stan  2.5.0  probability, sampling & optimization |


- Main Page
- Related Pages
- Namespaces
- Classes
- Files

- Class List
- Class Index
- Class Hierarchy
- Class Members

All Classes Namespaces Files Functions Variables Typedefs Enumerations Enumerator Friends Macros Pages

- stan
- gm
- write\_csv\_visgen

stan::gm::write\_csv\_visgen Member List

This is the complete list of members for stan::gm::write\_csv\_visgen, including all inherited members.

|  |  |  |
| --- | --- | --- |
| generate\_initialize\_array(const std::string &var\_type, const std::string &read\_type, const std::vector< expression > &read\_args, const std::string &name, const std::vector< expression > &dims) const | stan::gm::write\_csv\_visgen | inline |
| generate\_initialize\_array\_bounded(const D &x, const std::string &base\_type, const std::string &read\_fun\_prefix, const std::vector< expression > &dim\_args) const | stan::gm::write\_csv\_visgen | inline |
| o\_ | stan::gm::visgen |  |
| operator()(const nil &) const | stan::gm::write\_csv\_visgen | inline |
| operator()(const int\_var\_decl &x) const | stan::gm::write\_csv\_visgen | inline |
| operator()(const double\_var\_decl &x) const | stan::gm::write\_csv\_visgen | inline |
| operator()(const vector\_var\_decl &x) const | stan::gm::write\_csv\_visgen | inline |
| operator()(const row\_vector\_var\_decl &x) const | stan::gm::write\_csv\_visgen | inline |
| operator()(const matrix\_var\_decl &x) const | stan::gm::write\_csv\_visgen | inline |
| operator()(const unit\_vector\_var\_decl &x) const | stan::gm::write\_csv\_visgen | inline |
| operator()(const simplex\_var\_decl &x) const | stan::gm::write\_csv\_visgen | inline |
| operator()(const ordered\_var\_decl &x) const | stan::gm::write\_csv\_visgen | inline |
| operator()(const positive\_ordered\_var\_decl &x) const | stan::gm::write\_csv\_visgen | inline |
| operator()(const cholesky\_factor\_var\_decl &x) const | stan::gm::write\_csv\_visgen | inline |
| operator()(const cholesky\_corr\_var\_decl &x) const | stan::gm::write\_csv\_visgen | inline |
| operator()(const cov\_matrix\_var\_decl &x) const | stan::gm::write\_csv\_visgen | inline |
| operator()(const corr\_matrix\_var\_decl &x) const | stan::gm::write\_csv\_visgen | inline |
| result\_type typedef | stan::gm::visgen |  |
| visgen(std::ostream &o) | stan::gm::visgen | inline |
| write\_csv\_visgen(std::ostream &o) | stan::gm::write\_csv\_visgen | inline |


---

[ Stan Home Page ]

*© 2011–2014,
Stan Development Team.*
